# NRS2002-stratified body composition and laboratory characteristics in hospitalized cancer patients assessed by bioelectrical impedance analysis

**DOI:** 10.3389/fnut.2026.1842533

**Published:** 2026-06-24

**Authors:** Ping Zhong, Qiong Lai, Nan Luo, Ya Zou, Xueli Du, Ying Zhang

**Affiliations:** Department of Oncology, The Third People's Hospital of Chengdu, Chengdu, Sichuan, China

**Keywords:** bioelectrical impedance analysis, body composition, inflammation, nutritional risk, Nutritional Risk Screening 2002

## Abstract

**Objective:**

The body composition and laboratory characteristics underlying Nutritional Risk Screening 2002 (NRS2002)-defined nutritional risk in hospitalized patients with cancer remain insufficiently described.

**Methods:**

This single-center retrospective cross-sectional study included 998 hospitalized patients with cancer. Nutritional risk was defined as NRS2002 ≥3. Bioelectrical impedance analysis (BIA) parameters and laboratory indicators related to inflammation and nutritional status were compared across NRS2002 categories. Multivariable logistic regression was used to assess adjusted associations between key body composition parameters and nutritional risk after adjustment for age, sex, body mass index, and cancer group.

**Results:**

Across increasing NRS2002 categories, extracellular water-to-total body water ratio increased, whereas body mass index decreased. Among laboratory indicators, albumin and hemoglobin tended to decrease, whereas C-reactive protein (CRP) tended to increase. In multivariable analyses, albumin was inversely associated with nutritional risk in the model without CRP (OR = 0.958, 95% CI 0.928–0.989, *P* = 0.009), whereas log1p-transformed CRP was positively associated with nutritional risk in the model including CRP (OR = 1.311, 95% CI 1.146–1.499, *P* < 0.001). In contrast, skeletal muscle index, visceral fat area, phase angle, and extracellular water-to-total body water ratio were not significantly associated with nutritional risk after adjustment.

**Conclusion:**

Laboratory indicators related to inflammation and nutritional status showed more consistent associations with NRS2002-defined nutritional risk than single-time-point BIA-derived body composition parameters. BIA-derived parameters may provide complementary information on body composition, but their interpretation should consider clinical status and the limitations of single-time-point assessment.

## 1 Introduction

During disease progression and antitumor treatment, patients with cancer commonly experience reduced dietary intake, increased metabolic demands, and systemic inflammatory responses, which may clinically manifest as weight loss, muscle depletion, fatigue, and fluid retention. The coexistence of multiple risk factors during hospitalization makes nutritional risk assessment an essential component of routine clinical management. Previous studies have shown that malnutrition is associated with a higher risk of infection and other complications, as well as prolonged hospital stay (1, 2). Among hospitalized patients with cancer, poor nutritional status may also reduce treatment tolerance, increase the risk of treatment-related toxicity, delay recovery, and contribute to treatment interruption or dose modification. These effects may further influence the continuity and effectiveness of antitumor treatment, particularly in patients with a high disease burden (3, 4). Therefore, beyond nutritional risk screening, identifying differences in body composition and related physiological indicators among high-risk patients may help improve clinical assessment and guide subsequent management (5). Nutritional Risk Screening 2002 (NRS2002) has been widely used to screen for nutritional risk in hospitalized patients. However, NRS2002 mainly provides risk stratification and offers limited information on the body composition and physiological changes underlying high-risk status, which may vary considerably across patients (6). Further characterization of high-risk patients may therefore improve the interpretation of nutritional risk in clinical practice. Bioelectrical impedance analysis (BIA) is a bedside method for assessing body composition and provides indicators of muscle mass, fat mass, and fluid distribution, including skeletal muscle index (SMI), visceral fat area (VFA), extracellular water-to-total body water ratio (ECW/TBW), and phase angle (PhA), thereby helping to reveal the components underlying changes in body weight (7). Previous studies have reported associations between BIA-derived indicators and nutritional status or clinical outcomes in different cancer populations (8). However, in hospitalized patients with cancer, the distribution of BIA parameters across different NRS2002 categories, as well as their relationship with common laboratory markers such as albumin, C-reactive protein (CRP), and hemoglobin, has not been clearly described and compared within the same cohort. In addition, even among patients with high NRS2002 scores, nutritional problems may present differently. Some patients mainly show inadequate intake and weight loss, whereas others present more prominent inflammation or fluid imbalance (9). Against this background, jointly describing BIA-derived body composition and laboratory indicators within the NRS2002 framework may help translate risk scores into more clinically interpretable features. Therefore, this study included hospitalized patients with cancer to systematically describe the distribution patterns and trends of BIA parameters and laboratory indicators related to inflammation and nutritional status across NRS2002 categories.

## 2 Methods and materials

### 2.1 Study design and participants

This was a single-center retrospective cross-sectional study. Data were obtained from the electronic medical records and the BIA body composition database of hospitalized patients in the Department of Medical Oncology at Chengdu Third People's Hospital between 2023 and 2025. The source population comprised patients admitted to the Department of Medical Oncology during the study period. In routine inpatient practice, NRS2002 screening and BIA assessment were performed for hospitalized patients. BIA was not completed in a small number of patients when electrode placement was not feasible because of skin lesions or other local conditions. All patients who met the inclusion and exclusion criteria and had both assessments available during the study period were included in the analysis. The inclusion criteria were as follows: (1) age ≥18 years; (2) pathologically confirmed malignant tumor; (3) completion of BIA assessment during hospitalization; and (4) completion of NRS2002 assessment during hospitalization. The exclusion criteria were as follows: (1) an interval of more than 2 days between the BIA assessment and NRS2002 evaluation, to ensure that both assessments reflected a similar clinical condition; and (2) missing NRS2002 total score or key BIA parameters, precluding the definition of nutritional risk or major study variables. For patients with multiple BIA measurements, only the record closest in time to the NRS2002 assessment was retained. The collected data included demographic characteristics, tumor-related information, comorbidities and lifestyle factors, nutritional risk assessment results, BIA parameters, and laboratory test results. The study was approved by the Ethics Committee of Chengdu Third People's Hospital (approval No. 2026-S-46).

### 2.2 Data collection

Data preprocessing mainly included variable cleaning, uniform coding, and analytical transformation. Tumor diagnosis text was first standardized and mapped into grouped categories. Because nutritional risk may differ according to tumor origin, particularly between gastrointestinal and non-gastrointestinal cancers, cancer types were grouped according to the primary tumor site. Different diagnostic expressions referring to the same anatomical site were merged into the same category, and cancer types with low frequencies were classified as “other” to maintain model stability and avoid sparse categories in regression analyses. Binary variables, including hypertension, coronary heart disease, diabetes mellitus, smoking, alcohol consumption, and history of cancer-related surgery, were coded as 0/1, and “not recorded” was treated as missing. Continuous variables were checked for units and plausible ranges, and extreme values were retained to reflect the real-world distribution of the data. Scale transformations were applied to selected variables, with ECW/TBW modeled per 0.01 increase and VFA per 10 cm^2^ increase. Because CRP showed a right-skewed distribution, log1p transformation, defined as log(CRP + 1), was applied in the regression analyses to reduce skewness and the influence of extreme values. Missing data were summarized, and missingness in key variables was reported. Among laboratory indicators, CRP had a relatively high proportion of missing values because it was measured according to clinical judgment or inflammatory assessment needs rather than routinely measured for all hospitalized patients. Therefore, missing CRP values mainly reflected routine clinical testing practice. The main analyses were based on complete-case datasets, with each analysis including only individuals with complete data on all variables required for that specific model. Because some laboratory indicators were missing, the actual sample size varied across models, and the number of included participants was reported for each regression model. No single imputation was performed to avoid introducing additional assumptions.

### 2.3 Bioelectrical impedance analysis measurement

BIA was performed using a PCA-550 body composition analyzer (Sichuan, China), a multi-frequency bioelectrical impedance device operating at 1, 5, 50, 250, 500, and 1,000 kHz. The device used an eight-point contact electrode method according to the manufacturer's instructions. Measurements were performed by trained nurses following a standardized protocol. All assessments were conducted in the morning after overnight fasting and before breakfast. Patients were assessed in the supine position after resting quietly for 30 min before measurement. Before the assessment, patients were asked to remove metal accessories and remain still during measurement. To reduce the potential influence of recent food intake and fluid infusion, BIA assessment was performed before major intravenous fluid administration whenever possible. The device was regularly calibrated and maintained according to the manufacturer's recommendations and hospital equipment-management procedures. Because this was a retrospective study based on routine inpatient practice, the timing of BIA assessment was not standardized according to chemotherapy cycle or treatment phase.

### 2.4 Primary outcome and category definitions

The primary outcome was nutritional risk status, defined according to the total NRS2002 score. NRS2002 ranges from 0 to 7, with higher scores indicating greater nutritional risk. In the main analyses, nutritional risk was defined as a binary outcome, with NRS2002 ≥3 indicating nutritional risk (NRS ≥3) and NRS2002 < 3 indicating no nutritional risk (NRS < 3). In addition, NRS2002 was categorized into three groups, 0–2, 3–4, and ≥5, for supplementary descriptive and trend analyses to examine whether body composition and laboratory indicators showed graded patterns across increasing NRS2002 scores.

### 2.5 Nutritional risk stratification and characterization of body composition and laboratory indicators

Based on NRS2002 stratification, BIA parameters and laboratory indicators related to inflammation and nutritional status were characterized across risk categories. These indicators covered muscle status, fluid distribution, fat distribution, and overall cellular status. Albumin, CRP, hemoglobin, and other laboratory indicators were incorporated to compare body composition and laboratory characteristics across different levels of nutritional risk.

### 2.6 Multivariable models and assessment of independent associations

With NRS ≥3 as the outcome, multivariable logistic regression was used to estimate the adjusted associations between key body composition parameters and nutritional risk. Prespecified potential confounders, including age, sex, BMI, and cancer group, were included in the models, together with the core body composition parameters. Cancer group was included to partially account for cancer-type heterogeneity. Covariates were selected based on clinical relevance, previous literature, and availability in routine clinical records, rather than by data-driven stepwise selection. The BIA-derived parameters were selected to represent major body composition domains, including skeletal muscle mass, visceral adiposity, fluid distribution, and phase angle-related cellular status. Other device-derived outputs were not entered simultaneously to avoid model redundancy, because many BIA parameters are closely interrelated. Given the role of inflammatory markers in nutritional risk and their missingness patterns, two main models were constructed. Model A included albumin and the neutrophil-to-lymphocyte ratio (NLR) to represent nutritional and inflammatory status. Model B further included transformed CRP on the basis of Model A to reflect inflammatory burden and to assess the stability of the estimated associations for key body composition parameters after additional adjustment for CRP.

### 2.7 Robustness and heterogeneity analyses

Sensitivity analyses were performed based on the two main models to examine the robustness of the findings to model specification. An additional variable for any comorbidity, defined as the presence of hypertension, diabetes mellitus, or coronary heart disease, was introduced into the main models, and hemoglobin was further added to evaluate changes in the estimated effects of key body composition parameters. Standardized effect-size models were also fitted, with effects expressed per 1-standard-deviation increase, to compare the relative magnitudes of associations across different indicators. Potential non-linear associations between key continuous indicators and nutritional risk were examined using restricted cubic splines to assess departures from linearity and identify possible threshold effects. Heterogeneity analyses were conducted across tumor system categories using stratified analyses and interaction tests to evaluate whether the associations between key indicators and nutritional risk were consistent across subgroups.

### 2.8 Statistical analysis

Given the retrospective cross-sectional design, all eligible patients with both NRS2002 assessment and BIA measurement during the study period were included, and no prespecified target sample size was set. All statistical analyses were performed using R. Continuous variables are presented as median (interquartile range), and categorical variables as number (percentage). For comparisons of baseline characteristics and key indicators between the two NRS2002 groups, the Wilcoxon rank-sum test was used for continuous variables, and the chi-square test or Fisher's exact test for categorical variables. To evaluate the potential impact of CRP missingness, baseline characteristics were compared between patients with and without available CRP data using the same statistical tests. Standardized mean differences were also calculated to assess between-group imbalance. Across the three NRS2002 categories (0–2, 3–4, and ≥5), the distributions of key body composition and laboratory indicators were described and their gradient patterns were assessed. Group comparisons were performed using the Kruskal–Wallis test, and trend analysis was conducted by coding the categories as 1, 2, and 3 and testing for a linear trend. Multivariable logistic regression was used with NRS ≥3 as the outcome, and odds ratios (ORs) with 95% confidence intervals (CIs) were reported. Multicollinearity in the multivariable models was assessed using variance inflation factors (VIFs), with VIF values < 5 considered to indicate no substantial multicollinearity. Robustness analyses included extended models with additional adjustment for comorbidity burden and hemoglobin, as well as standardized models expressing effects per 1-standard-deviation increase. Non-linear associations were examined using restricted cubic splines with three knots, and fitted curves were plotted. Heterogeneity was assessed across tumor system categories using stratified analyses and interaction tests, with *P* values for interaction reported. Model discrimination was additionally described using the area under the receiver operating characteristic curve (AUC), with 95% CIs calculated by the DeLong method. All tests were two-sided, and *P* < 0.05 was considered statistically significant.

## 3 Result

### 3.1 Study population and baseline characteristics

A total of 998 hospitalized patients with cancer were included in the study. According to the binary classification of NRS2002, 369 patients (37.0%) were classified as NRS < 3 and 629 (63.0%) as NRS ≥3. Key variables showed varying degrees of missingness, including 44 cases for SMI, 48 for ECW/TBW, 47 for VFA, 44 for PhA, 49 for BMI, 71 for albumin, and 143 for CRP. The sex distribution was similar between the two groups (*P* = 0.310), whereas the distribution of tumor system categories differed significantly (*P* = 0.020). Compared with the NRS < 3 group, the NRS ≥3 group was older (69.0 [60.0, 76.0] vs. 61.0 [55.0, 71.0], *P* < 0.001). Among body composition parameters, ECW/TBW was higher in the NRS ≥3 group (0.39 [0.38, 0.40] vs. 0.38 [0.38, 0.39], *P* < 0.001), whereas BMI, SMI, VFA, and PhA did not differ significantly between groups (all *P* > 0.05). For laboratory indicators, the NRS ≥3 group had lower albumin levels (40.00 [36.30, 43.12] g/L vs. 40.70 [37.90, 43.35] g/L, *P* = 0.009), higher CRP levels (10.76 [3.23, 44.00] mg/L vs. 6.82 [2.21, 23.22] mg/L, *P* < 0.001), and lower hemoglobin levels (130 [115, 142] g/L vs. 133 [120, 144] g/L, *P* = 0.006), while NLR did not differ significantly between groups (*P* = 0.131). No significant between-group differences were observed in hypertension, coronary heart disease, diabetes mellitus, smoking, alcohol consumption, or history of cancer-related surgery (all *P* > 0.05) (Table 1). Given the relatively high proportion of missing CRP values, a supplementary comparison was performed between patients with and without available CRP data. As shown in Supplementary Table 1, patients with available CRP data had a higher proportion of nutritional risk than those without CRP data, and differences were also observed in sex distribution, smoking, and alcohol consumption.

**Table 1:** Baseline characteristics stratified by nutritional risk (NRS2002 <3 vs. ≥3).

Variable	NRS < 3 (*n* = 369)	NRS ≥3 (*n* = 629)	*P* value
Sex			0.310
Female	182 (49.3%)	288 (45.8%)	
Male	187 (50.7%)	341 (54.2%)	
Cancer group			0.020
Breast & Gynecologic	65 (17.6%)	71 (11.3%)	
Gastrointestinal	189 (51.2%)	375 (59.6%)	
Genitourinary	17 (4.6%)	16 (2.5%)	
Head & Neck	12 (3.3%)	31 (4.9%)	
Hematologic	2 (0.5%)	5 (0.8%)	
Other	14 (3.8%)	25 (4.0%)	
Thoracic	70 (19.0%)	106 (16.9%)	
Hypertension			0.107
No	256 (69.8%)	406 (64.5%)	
Yes	111 (30.2%)	223 (35.5%)	
Coronary artery disease			0.281
No	342 (92.9%)	569 (90.7%)	
Yes	26 (7.1%)	58 (9.3%)	
Diabetes mellitus			0.578
No	307 (83.4%)	513 (81.8%)	
Yes	61 (16.6%)	114 (18.2%)	
Smoking			0.955
No	249 (67.5%)	422 (67.1%)	
Yes	120 (32.5%)	207 (32.9%)	
Alcohol use			0.793
No	281 (76.2%)	473 (75.2%)	
Yes	88 (23.8%)	156 (24.8%)	
Cancer-related surgery			0.558
No	186 (50.5%)	304 (48.4%)	
Yes	182 (49.5%)	324 (51.6%)	
Age, years	61.00 [55.00, 71.00]	69.00 [60.00, 76.00]	< 0.001
BMI, kg/m^2^	22.00 [20.10, 24.20]	21.80 [19.58, 24.00]	0.111
SMI, kg/m^2^	7.20 [6.40, 8.10]	7.20 [6.30, 8.10]	0.521
ECW/TBW	0.38 [0.38, 0.39]	0.39 [0.38, 0.40]	< 0.001
VFA, cm^2^	46.10 [29.90, 65.80]	47.20 [31.00, 68.97]	0.501
PhA, °	5.40 [5.00, 5.80]	5.30 [4.90, 5.80]	0.204
Albumin, g/L	40.70 [37.90, 43.35]	40.00 [36.30, 43.12]	0.009
CRP, mg/L	6.82 [2.21, 23.22]	10.76 [3.23, 44.00]	< 0.001
Hemoglobin, g/L	133 [120, 144]	130 [115, 142]	0.006
NLR	3.03 [2.12, 4.70]	3.35 [2.15, 5.40]	0.131

Categorical variables are presented as *n* (%), and continuous variables as median [IQR]. Owing to missing data, comparisons were based on available cases for each variable; therefore, denominators may vary across rows. The total study population included 998 patients.

### 3.2 Body composition and laboratory characteristics across NRS2002 categories

**Figure 1:**
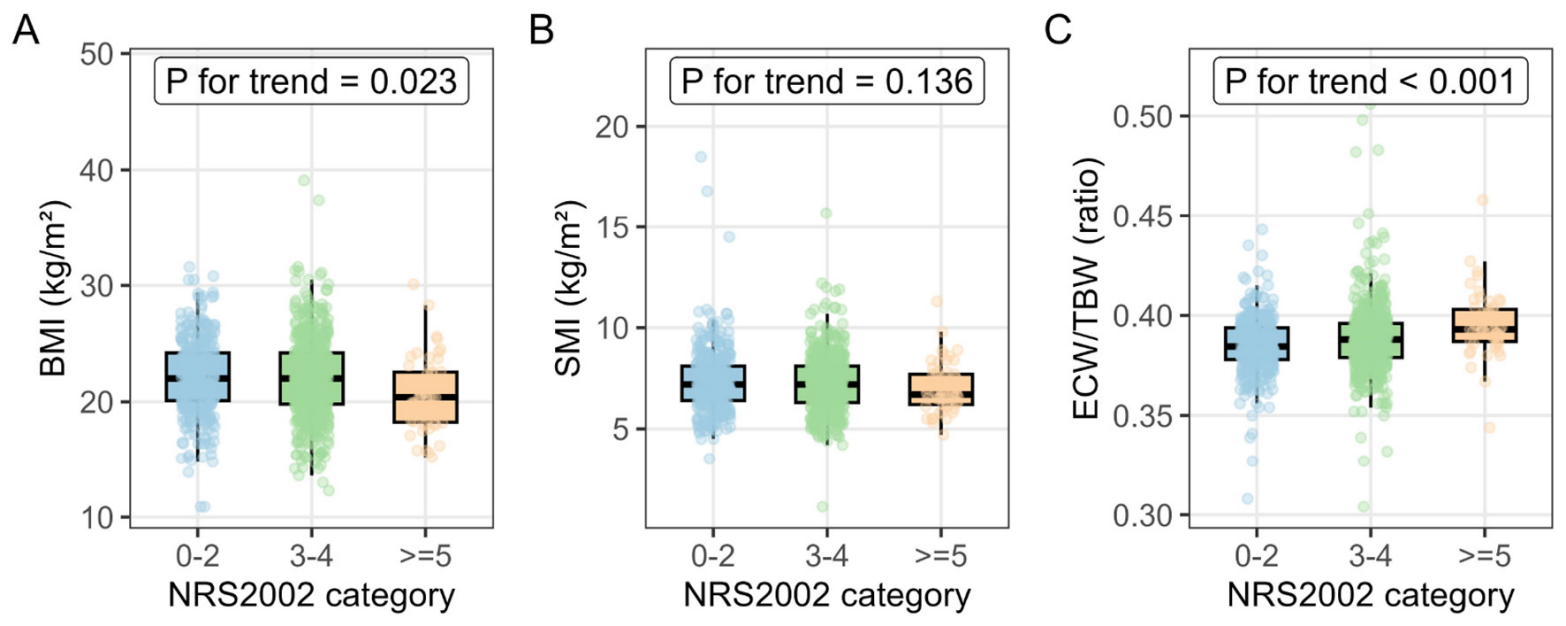
Key body composition indicators across NRS2002 categories. Boxplots show the distribution of **(A)** BMI, **(B)** SMI, and **(C)** ECW/TBW across NRS2002 categories (0–2, 3–4, ≥5). Center lines indicate medians, boxes indicate interquartile ranges, and whiskers indicate 1.5× IQR. Points represent individual observations. *P* values denote *P* for trend across ordered NRS2002 categories.

**Figure 2:**
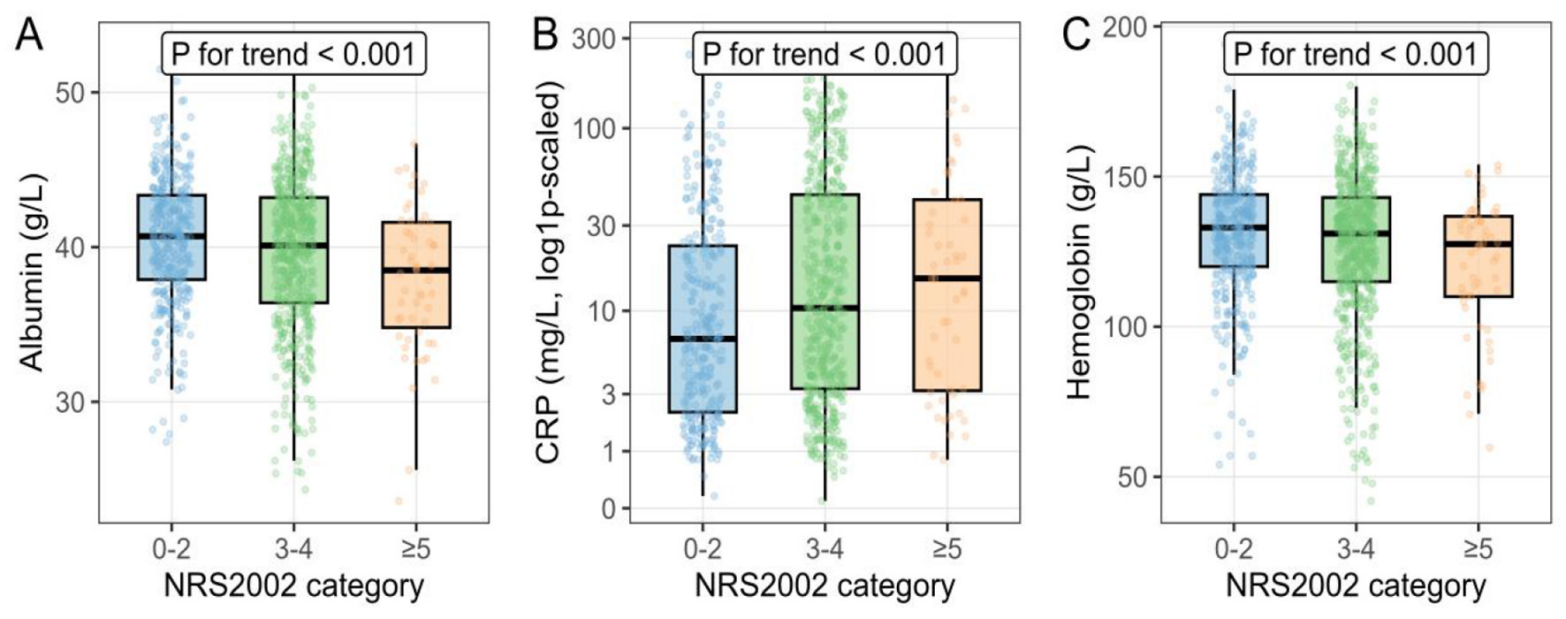
Key laboratory indicators across NRS2002 categories. Boxplots show the distribution of **(A)** albumin, **(B)** C-reactive protein (CRP), and **(C)** hemoglobin across NRS2002 categories (0–2, 3–4, ≥5). Center lines indicate medians, boxes indicate interquartile ranges, and whiskers indicate 1.5× IQR. Points represent individual observations. CRP values are expressed in mg/L, and a log1p-scaled y-axis was used only for visualization because of the right-skewed distribution. *P* values denote *P* for trend across ordered NRS2002 categories.

Across the NRS2002 categories (0–2, 3–4, and ≥5), some body composition and laboratory indicators showed gradient patterns. ECW/TBW increased progressively with higher NRS2002 categories (*P* for trend < 0.001), whereas BMI decreased with increasing risk level (*P* for trend = 0.023). No significant trend was observed for SMI (*P* for trend = 0.136) (Figure 1). Among laboratory indicators, albumin and hemoglobin decreased progressively with increasing NRS2002 categories (both *P* for trend < 0.001), whereas CRP increased progressively (*P* for trend < 0.001) (Figure 2). The distributions and trend analyses of the remaining body composition and laboratory indicators across the three NRS2002 categories are presented in Supplementary Table 2.

### 3.3 Multivariable associations with nutritional risk

After adjustment for age, sex, BMI, and cancer group, multivariable logistic regression was used to evaluate the adjusted associations between key body composition parameters and nutritional risk (NRS ≥3). Complete-case analysis was applied. Because CRP had a relatively high proportion of missing values, Model B, which included CRP, had a smaller effective sample size than Model A. In Model A (albumin + NLR; *n* = 876), albumin was inversely associated with nutritional risk (OR = 0.958, 95% CI 0.928–0.989, *P* = 0.009), whereas no significant associations were observed for SMI, ECW/TBW, VFA, PhA, or NLR (all *P* > 0.05). In Model B, which further included log1p-transformed CRP on the basis of Model A (*n* = 752), log1p(CRP) was positively associated with nutritional risk (OR = 1.311, 95% CI 1.146–1.499, *P* < 0.001), whereas albumin was no longer statistically significant. None of the body composition parameters showed a significant adjusted association with nutritional risk in this model (all *P* > 0.05) (Table 2). In Model B, the VIF values for albumin and log1p(CRP) were 1.073 and 1.095, respectively, and all VIF values were below 5, indicating no substantial multicollinearity.

**Table 2:** Multivariable logistic regression analysis of factors associated with nutritional risk (NRS2002 ≥3).

Predictor	Model A OR (95% CI)	*P* value	Model B OR (95% CI)	*P* value
Age (per 1 year)	1.035 (1.022–1.048)	< 0.001	1.040 (1.025–1.055)	< 0.001
Male sex (vs. female)	0.950 (0.675–1.338)	0.771	0.840 (0.571–1.234)	0.373
BMI (per 1 kg/m^2^)	0.934 (0.873–0.999)	0.048	0.931 (0.864–1.002)	0.056
Cancer group: Gastrointestinal	1.591 (0.991–2.554)	0.055	1.728 (1.018–2.932)	0.043
Cancer group: Genitourinary	0.661 (0.260–1.676)	0.383	0.990 (0.352–2.785)	0.985
Cancer group: Head & Neck	2.680 (1.142–6.290)	0.023	3.652 (1.418–9.406)	0.007
Cancer group: Hematologic	1.586 (0.279–9.029)	0.603	1.696 (0.292–9.842)	0.556
Cancer group: Other	1.572 (0.696–3.553)	0.277	2.141 (0.840–5.456)	0.111
Cancer group: Thoracic	1.005 (0.573–1.763)	0.985	1.267 (0.673–2.386)	0.463
SMI (per 1 kg/m^2^)	1.032 (0.916–1.163)	0.608	0.984 (0.857–1.131)	0.824
ECW/TBW (per 0.01 increase)	1.052 (0.954–1.159)	0.313	1.045 (0.938–1.164)	0.427
VFA (per 10 cm^2^ increase)	1.056 (0.986–1.132)	0.117	1.064 (0.987–1.148)	0.106
Phase angle (per 1°)	1.016 (0.971–1.063)	0.492	1.121 (0.942–1.333)	0.198
Albumin (per 1 g/L)	0.958 (0.928–0.989)	0.009	0.982 (0.947–1.019)	0.343
NLR (per 1 unit)	0.998 (0.984–1.013)	0.817	0.984 (0.951–1.018)	0.359
log1p(CRP)	—	—	1.311 (1.146–1.499)	< 0.001

Values are odds ratios (ORs) with 95% confidence intervals (CIs). Model A included age, sex, BMI, cancer group, SMI, ECW/TBW, VFA, phase angle, albumin, and NLR. Model B further included log1p-transformed CRP on the basis of Model A. The reference group for cancer group was breast and gynecologic cancers. ECW/TBW was modeled per 0.01 increase, and VFA was modeled per 10 cm^2^ increase. CRP, C-reactive protein; NLR, neutrophil-to-lymphocyte ratio; SMI, skeletal muscle index; ECW/TBW, extracellular water-to-total body water ratio; VFA, visceral fat area.

To address the difference in analytic samples between models with and without CRP, Model A was additionally refitted in the CRP-complete subset. Albumin remained inversely associated with nutritional risk in the CRP-complete subset before CRP adjustment, whereas this association was attenuated after further adjustment for log1p-transformed CRP in Model B. Log1p-transformed CRP remained positively associated with nutritional risk. These findings suggest that differences between Model A and Model B should be interpreted in the context of both CRP adjustment and CRP data availability (Supplementary Table 3).

### 3.4 Sensitivity analyses and supplementary findings

Sensitivity analyses showed that the main findings were materially unchanged after further adjustment for comorbidity burden or hemoglobin. Albumin remained inversely associated with nutritional risk in Model A and its extended models, whereas log1p(CRP) remained positively associated with nutritional risk in all models that included CRP (Supplementary Table 4). Reanalysis using standardized variables expressed per 1-standard-deviation increase yielded results consistent with the main analyses (Supplementary Table 5). Restricted cubic spline analysis suggested a non-linear association between albumin and nutritional risk, and the corresponding curve is shown in Supplementary Figure 1. Interaction analyses did not identify significant effect modification by GI vs. non-GI grouping for the body composition parameters; the interaction term between log1p(CRP) and GI was also not statistically significant (*P* = 0.090) (Supplementary Table 6). As a descriptive supplementary measure of model discrimination, the AUC was 0.658 (95% CI 0.621–0.696) for Model A and 0.679 (95% CI 0.639–0.719) for Model B.

## 4 Discussion

In this single-center study of nearly 1,000 hospitalized patients with cancer, we compared body composition and laboratory characteristics across NRS2002-defined nutritional risk categories using BIA and routine laboratory data. Across increasing NRS2002 categories, ECW/TBW and several laboratory indicators showed statistically significant but generally modest differences. Albumin and hemoglobin tended to be lower, whereas CRP tended to be higher, in patients with higher NRS2002 scores. After adjustment for age, sex, BMI, and cancer group, multivariable logistic regression showed that log1p(CRP) was positively associated with nutritional risk in models including CRP, whereas albumin was inversely associated with nutritional risk in models without CRP. However, the interpretation of CRP-related models requires caution because CRP was not uniformly available. In contrast, SMI, VFA, PhA, and ECW/TBW were not significantly associated with nutritional risk after adjustment. Overall, inflammatory indicators appeared to show more consistent associations with NRS2002-defined nutritional risk, whereas single-time-point BIA-derived body composition parameters did not demonstrate stable independent associations after multivariable adjustment.

From the perspective of inflammation and disease burden, NRS2002 is intended to identify patients at risk who may require nutritional support, and its score incorporates both the degree of nutritional impairment and disease severity. Accordingly, higher NRS2002 scores often coexist with greater disease burden and more active inflammation (10). CRP reflects inflammatory burden, and inflammation is commonly accompanied by increased metabolic demand, reduced appetite, and inadequate intake, all of which may contribute to higher NRS2002 scores (11, 12). By contrast, although albumin is widely used in clinical nutritional assessment, it is strongly influenced by inflammation and fluid status and may reflect disease activity and the acute-phase response rather than inadequate intake alone (13, 14). In the present study, the association between albumin and nutritional risk was attenuated after additional adjustment for log1p(CRP), which may suggest that albumin and CRP partly reflect overlapping inflammation-related information. However, this finding should be interpreted cautiously because CRP was not uniformly measured and CRP availability was not completely random. In contrast, some body composition indicators differed across NRS2002 categories but did not show stable associations after adjustment for age, BMI, and cancer group. This finding suggests that their relationship with nutritional risk may be more susceptible to differences in population composition and inflammatory status. Previous studies have shown that BIA-derived estimates of muscle mass and PhA are influenced by changes in fluid distribution (15). In populations in which extracellular fluid expansion or hypoalbuminemia is more common, the interpretability and stability of body composition indicators may therefore be reduced (16). This may partly explain why single-time-point BIA-derived body composition parameters did not show stable cross-sectional associations with NRS2002-defined nutritional risk in this heterogeneous hospitalized cohort, rather than indicating that these parameters are biologically or clinically irrelevant. From a clinical perspective, inflammatory indicators and BIA-derived body composition parameters may be better viewed as complementary sources of information in nutritional risk assessment. Inflammatory markers primarily reflect inflammatory burden and physiological stress, whereas body composition indicators provide additional information on body habitus and composition, such as low muscle mass, reduced fat reserve, or abnormal fluid distribution. However, these indicators should be interpreted together with NRS2002 and other clinical information rather than used as standalone indicators of nutritional risk. Together, they may help identify the focus of nutritional intervention and guide follow-up monitoring (17, 18). In the present study, ECW/TBW increased with higher NRS2002 categories, although the absolute difference was modest, suggesting that abnormal fluid distribution may be more common among patients at higher nutritional risk. ECW/TBW is commonly regarded as an indicator of fluid retention or volume overload. Previous studies have shown that ECW/TBW is associated with adverse constitutional states such as cachexia, sarcopenia, and frailty (19, 20), suggesting that BIA-based assessment of fluid distribution may provide complementary information for nutritional risk evaluation in hospitalized patients with cancer (21). Although ECW/TBW and BMI differed at the stratified descriptive level, neither showed a stable association after multivariable adjustment. This finding suggests that their relationship with nutritional risk may be influenced by age, body size, cancer group, and inflammatory status, which is broadly consistent with previous reports (22, 23).

SMI, VFA, and PhA are important indicators for evaluating body composition and nutritional status. As a height-adjusted skeletal muscle measure, SMI is an important marker for identifying muscle loss and malnutrition (24, 25). According to GLIM-related criteria, one study found that approximately one quarter of patients classified as not at nutritional risk by NRS2002 still had low SMI (26), indicating that NRS2002 scores do not fully correspond to low-muscle-mass malnutrition. In the present cross-sectional cohort, SMI did not clearly discriminate NRS2002-defined nutritional risk in either stratified analyses or adjusted models. This may be because NRS2002 integrates recent intake, weight change, and disease severity, whereas muscle mass changes relatively slowly and a single measurement may not correspond closely to the risk score assessed at hospitalization. VFA reflects visceral fat reserve and fat distribution (27). Importantly, the relationship between VFA and nutritional risk is not necessarily unidirectional. Depending on the disease context, treatment stage, and metabolic status, VFA may increase or decrease; therefore, low VFA should not be interpreted simply as high nutritional risk, nor should high VFA be interpreted as favorable nutritional status (28). In this cross-sectional cohort, VFA did not show a clear linear trend across NRS2002 categories and was not stably associated with nutritional risk after adjustment for age, sex, BMI, and cancer group. This is broadly consistent with previous findings suggesting that visceral fat area may not show a simple cross-sectional correspondence with NRS2002-defined nutritional risk in hospitalized patients with cancer (29). Therefore, in patients with cancer, NRS2002 and VFA-related body composition indicators may be better viewed as complementary tools in nutritional risk assessment (30). PhA has been regarded as a convenient indicator of nutritional status and a potential biomarker of adverse outcomes in several conditions, including cancer (31), cirrhosis (32), and hemodialysis populations (33). Previous studies have suggested that higher nutritional risk is often associated with lower PhA (34), and in hospitalized older adults and some cancer populations, PhA has shown a certain ability to distinguish patients identified by NRS2002 as being at nutritional risk (35, 36). In the present cross-sectional cohort, PhA did not show clear differences across NRS2002 categories or stable associations with NRS ≥3 after adjustment. This result may reflect the heterogeneity of tumor types and treatment stages, variability in inflammatory and nutritional status, and the influence of acute clinical conditions on BIA measurements. Therefore, the null finding in this study should not be taken as evidence that PhA lacks clinical value. Previous longitudinal and prognostic studies have suggested that PhA may be more informative for monitoring changes in nutritional or functional status and for assessing adverse outcomes than for single-time-point discrimination of nutritional risk (37, 38). Further research and clinical application should continue to define the appropriate use and interpretive boundaries of PhA.

This study has several strengths. First, the sample size was relatively adequate and included patients across multiple tumor system categories. Second, within a unified NRS2002-based risk framework, we integrated BIA-derived body composition parameters with laboratory indicators related to inflammation and nutritional status for stratified description and multivariable analysis. We further supported the main findings through robustness analyses, standardized models, interaction tests, and restricted cubic spline analyses. Nevertheless, several limitations should be acknowledged. First, this was a single-center retrospective cross-sectional study, and the findings reflect associations at the time of hospitalization rather than causal relationships. Second, although cancer group was included as an adjustment variable, several clinically important factors, including cancer stage, current treatment modality, and functional or performance status, were not uniformly available in the retrospective dataset and therefore could not be incorporated into the primary multivariable models. These factors may influence nutritional risk, inflammatory markers, and body composition; thus, residual confounding related to disease severity, treatment status, and functional status cannot be excluded. Third, most patients underwent only a single BIA assessment, and body composition estimates may have been influenced by abnormalities in fluid distribution. Recent use of diuretics or other interventions affecting fluid distribution was not uniformly recorded and therefore could not be fully controlled. Intra- and inter-observer reliability was not formally assessed because the study was based on retrospective routine clinical data. Another limitation is that some laboratory indicators, particularly CRP, were missing because they were not uniformly measured in routine inpatient practice, resulting in different effective sample sizes across models. The supplementary comparison suggested that CRP availability was not completely random; therefore, potential bias related to missing data and selective CRP testing cannot be fully excluded, and CRP-related analyses should be interpreted cautiously. Future multicenter studies incorporating repeated longitudinal measurements and more comprehensive clinical assessment are needed to validate these findings and to further explore their value in clinical nutritional management.

## 5 Conclusion

Within the NRS2002 stratification framework, this study described the distributional characteristics of body composition and laboratory indicators in hospitalized patients with cancer and further showed that, after adjustment for common confounders, inflammatory burden-related indicators had more stable associations with nutritional risk. From the perspective of clinical nutrition practice, these findings suggest that nutritional risk assessment and intervention decisions should not rely solely on a single body composition indicator, but should instead incorporate information on inflammatory status, fluid distribution, and body habitus. Future multicenter studies incorporating repeated longitudinal measurements, information on dietary intake and weight change, and functional outcomes are warranted to further clarify the temporal relationships among body composition, inflammation, and nutritional risk, and to explore the feasibility of integrating BIA-derived indicators into nutritional management pathways.

## Data Availability

The original contributions presented in the study are included in the article and Supplementary material. Further inquiries can be directed to the corresponding author.
