# Supplementary material for: NRS2002-stratified body composition and laboratory characteristics in hospitalized cancer patients assessed by bioelectrical impedance analysis
Source: Front Nutr. 2026 Jun 24;13:1842533. doi: 10.3389/fnut.2026.1842533 (PMC13341418; doi:10.3389/fnut.2026.1842533)
Supplement: Supplementary file 1 [file Table_1.docx]

**Supplementary Table S1. Baseline characteristics of patients with and without available CRP data**

| Variable |  | CRP available (n = 855) | CRP missing (n = 143) | P value | SMD |
| --- | --- | --- | --- | --- | --- |
| Age, years |  | 67.00 [58.00, 74.00] | 64.00 [55.00, 73.00] | 0.117 | 0.184 |
| BMI, kg/m² |  | 21.90 [19.80, 24.13] | 22.00 [19.70, 23.90] | 0.556 | 0.089 |
| NRS2002 total score |  | 3.00 [2.00, 4.00] | 2.00 [2.00, 3.00] | <0.001 | 0.346 |
| SMI, kg/m² |  | 7.20 [6.30, 8.10] | 7.20 [6.30, 8.00] | 0.617 | 0.112 |
| ECW/TBW |  | 0.39 [0.38, 0.40] | 0.39 [0.38, 0.39] | 0.393 | 0.124 |
| VFA, cm² |  | 46.60 [31.02, 68.25] | 44.30 [27.40, 62.50] | 0.321 | 0.121 |
| Phase angle |  | 5.40 [4.90, 5.70] | 5.40 [5.00, 5.80] | 0.263 | 0.142 |
| Albumin, g/L |  | 40.20 [36.90, 43.30] | 40.60 [36.55, 42.55] | 0.474 | 0.070 |
| Hemoglobin, g/L |  | 132.00 [116.00, 144.00] | 129.00 [117.00, 137.50] | 0.184 | 0.047 |
| NLR |  | 3.16 [2.14, 5.09] | 3.28 [2.00, 5.07] | 0.744 | 0.182 |
| Sex | Female | 391 (45.7%) | 79 (55.2%) | 0.043 | 0.190 |
|  | Male | 464 (54.3%) | 64 (44.8%) |  |  |
| Cancer group | Breast & Gynecologic | 113 (13.2%) | 23 (16.1%) |  | 0.080 |
|  | Gastrointestinal | 493 (57.7%) | 71 (49.7%) |  |  |
|  | Genitourinary | 27 (3.2%) | 6 (4.2%) |  |  |
|  | Head & Neck | 39 (4.6%) | 4 (2.8%) |  |  |
|  | Hematologic | 7 (0.8%) | 0 (0.0%) |  |  |
|  | Other | 31 (3.6%) | 8 (5.6%) |  |  |
|  | Thoracic | 145 (17.0%) | 31 (21.7%) |  |  |
| Hypertension | No | 565 (66.1%) | 97 (67.8%) | 0.252 | 0.046 |
|  | Yes | 289 (33.8%) | 45 (31.5%) |  |  |
|  | Missing | 1 (0.1%) | 1 (0.7%) |  |  |
| Coronary heart disease | No | 774 (90.5%) | 137 (95.8%) | 0.120 | 0.199 |
|  | Yes | 78 (9.1%) | 6 (4.2%) |  |  |
|  | Missing | 3 (0.4%) | 0 (0.0%) |  |  |
| Diabetes mellitus | No | 695 (81.3%) | 125 (87.4%) | 0.176 | 0.161 |
|  | Yes | 157 (18.4%) | 18 (12.6%) |  |  |
|  | Missing | 3 (0.4%) | 0 (0.0%) |  |  |
| Smoking | No | 562 (65.7%) | 109 (76.2%) | 0.017 | 0.231 |
|  | Yes | 293 (34.3%) | 34 (23.8%) |  |  |
| Alcohol consumption | No | 634 (74.2%) | 120 (83.9%) | 0.016 | 0.240 |
|  | Yes | 221 (25.8%) | 23 (16.1%) |  |  |
| History of cancer-related surgery | No | 414 (48.4%) | 76 (53.1%) | 0.502 | 0.092 |
|  | Yes | 439 (51.3%) | 67 (46.9%) |  |  |
|  | Missing | 2 (0.2%) | 0 (0.0%) |  |  |
| NRS2002 nutritional risk | NRS2002 <3 | 294 (34.4%) | 75 (52.4%) | <0.001 | 0.364 |
|  | NRS2002 ≥3 | 561 (65.6%) | 68 (47.6%) |  |  |
| NRS2002 category | 0–2 | 294 (34.4%) | 75 (52.4%) | <0.001 | 0.181 |
|  | 3–4 | 509 (59.5%) | 62 (43.4%) |  |  |
|  | ≥5 | 52 (6.1%) | 6 (4.2%) |  |  |

**Note:** SMD was used to assess between-group imbalance, with an SMD ≥0.10 indicating potential imbalance. Missing categories are shown where applicable.

**Supplementary Table S2. Distribution of body composition and laboratory indicators across NRS2002 categories**

| Variable | 0–2 | 3–4 | ≥5 | Non-missing n | Missing n | P for group difference | P for trend |
| --- | --- | --- | --- | --- | --- | --- | --- |
| **Body composition indicators** | | | | | | | |
| BMI | 22.00 [20.10, 24.20] | 22.00 [19.80, 24.20] | 20.40 [18.25, 22.55] | 949 | 49 | 0.003 | 0.023 |
| SMI | 7.20 [6.40, 8.10] | 7.20 [6.30, 8.10] | 6.70 [6.20, 7.70] | 954 | 44 | 0.286 | 0.136 |
| Phase angle | 5.40 [5.00, 5.80] | 5.30 [4.90, 5.80] | 5.30 [4.90, 5.70] | 954 | 44 | 0.221 | 0.358 |
| Visceral fat area | 46.10 [29.90, 65.80] | 48.20 [31.10, 70.20] | 37.70 [29.40, 58.50] | 951 | 47 | 0.180 | 0.673 |
| ECW/TBW | 0.38 [0.38, 0.39] | 0.39 [0.38, 0.40] | 0.39 [0.39, 0.40] | 950 | 48 | <0.001 | <0.001 |
| Muscle mass | 41.90 [37.10, 47.90] | 42.10 [36.30, 47.90] | 39.90 [35.30, 44.00] | 954 | 44 | 0.163 | 0.722 |
| Body fat mass | 12.30 [7.80, 16.00] | 12.60 [7.65, 17.05] | 8.70 [4.80, 13.40] | 954 | 44 | 0.003 | 0.691 |
| Body fat percentage | 21.40 [14.50, 27.90] | 21.20 [14.10, 28.70] | 17.70 [9.90, 25.70] | 953 | 45 | 0.045 | 0.089 |
| Waist-to-hip ratio | 0.74 [0.70, 0.83] | 0.75 [0.69, 0.83] | 0.72 [0.68, 0.80] | 953 | 45 | 0.380 | 0.361 |
| Body water percentage | 57.50 [52.75, 62.77] | 57.80 [52.20, 62.90] | 60.40 [54.80, 65.80] | 954 | 44 | 0.021 | 0.059 |
| Basal metabolic rate | 1333.00 [1224.25, 1469.50] | 1334.00 [1201.00, 1463.75] | 1283.00 [1176.00, 1384.00] | 953 | 45 | 0.134 | 0.220 |
| Protein | 8.65 [7.60, 9.90] | 8.70 [7.40, 9.95] | 8.20 [7.30, 8.90] | 954 | 44 | 0.141 | 0.061 |
| Mineral | 3.00 [2.70, 3.40] | 3.00 [2.70, 3.50] | 2.90 [2.70, 3.20] | 954 | 44 | 0.443 | 0.647 |
| ECF/TBF | 0.34 [0.33, 0.35] | 0.34 [0.33, 0.35] | 0.34 [0.34, 0.35] | 954 | 44 | <0.001 | 0.727 |
| **Laboratory indicators** | | | | | | | |
| Albumin | 40.70 [37.90, 43.35] | 40.10 [36.40, 43.20] | 38.50 [34.80, 41.60] | 927 | 71 | 0.002 | <0.001 |
| C-reactive protein | 6.82 [2.21, 23.22] | 10.38 [3.27, 44.12] | 15.30 [3.17, 41.41] | 855 | 143 | <0.001 | <0.001 |
| Hemoglobin | 133.00 [120.00, 144.00] | 131.00 [115.00, 143.00] | 127.50 [110.00, 136.75] | 998 | 0 | 0.003 | <0.001 |
| NLR | 3.03 [2.12, 4.70] | 3.26 [2.15, 5.29] | 4.23 [2.34, 6.50] | 998 | 0 | 0.090 | 0.422 |
| Prealbumin | 230.20 [174.75, 274.55] | 216.40 [154.70, 260.80] | 188.30 [138.60, 239.35] | 727 | 271 | <0.001 | <0.001 |
| Total protein | 70.20 [65.38, 74.10] | 70.10 [65.68, 73.90] | 68.50 [63.65, 72.90] | 927 | 71 | 0.175 | 0.087 |
| Globulin | 29.20 [25.90, 32.10] | 29.30 [26.30, 32.80] | 29.30 [25.55, 32.80] | 927 | 71 | 0.501 | 0.298 |
| Albumin-to-globulin ratio | 1.40 [1.30, 1.60] | 1.40 [1.20, 1.50] | 1.30 [1.15, 1.50] | 930 | 68 | 0.010 | 0.003 |

Note: Values are presented as median [interquartile range]. Group differences across the three NRS2002 categories were assessed using the Kruskal–Wallis test. P for trend was calculated by coding NRS2002 categories as 1, 2, and 3 and testing for a linear trend. Non-missing and missing sample sizes are reported for each variable. NRS2002 categories were defined as 0–2, 3–4, and ≥5.

**Supplementary Table S3. Sensitivity analysis comparing Model A and Model B in the CRP-complete subset**

| **Variable** | **Model A, original complete-case sample n=876** | **Model A, CRP-complete subset n=752** | **Model B, CRP-complete subset n=752** |
| --- | --- | --- | --- |
| SMI | 1.032 (0.916, 1.163), P=0.608 | 0.971 (0.844, 1.117), P=0.681 | 0.984 (0.857, 1.131), P=0.824 |
| ECW/TBW, per 0.01 increase | 1.052 (0.954, 1.159), P=0.313 | 1.040 (0.934, 1.158), P=0.473 | 1.045 (0.938, 1.164), P=0.427 |
| VFA, per 10 cm² increase | 1.056 (0.986, 1.132), P=0.117 | 1.063 (0.987, 1.145), P=0.107 | 1.064 (0.987, 1.148), P=0.106 |
| Phase angle | 1.016 (0.971, 1.063), P=0.492 | 1.136 (0.939, 1.376), P=0.189 | 1.121 (0.942, 1.333), P=0.198 |
| Albumin | 0.958 (0.928, 0.989), P=0.009 | 0.963 (0.930, 0.998), P=0.036 | 0.982 (0.947, 1.019), P=0.343 |
| NLR | 0.998 (0.984, 1.013), P=0.817 | 1.001 (0.967, 1.036), P=0.955 | 0.984 (0.951, 1.018), P=0.359 |
| log1p(CRP) | - | - | 1.311 (1.146, 1.499), P<0.001 |

Note: Values are odds ratios with 95% confidence intervals. Model A included age, sex, BMI, cancer group, SMI, ECW/TBW, VFA, phase angle, albumin, and NLR. Model B was fitted in the CRP-complete subset and further included log1p-transformed CRP. ECW/TBW was modeled per 0.01 increase, and VFA was modeled per 10 cm² increase.

**Supplementary Table S4. Robustness analyses of multivariable models for nutritional risk (NRS2002 ≥3)**

| Predictor (unit) | Model A OR (95%CI) | p | Model A + comorb OR (95%CI) | p | Model B OR (95%CI) | p | Model B + comorb OR (95%CI) | p | Model B + Hb OR (95%CI) | p |
| --- | --- | --- | --- | --- | --- | --- | --- | --- | --- | --- |
| SMI (per 1 kg/m²) | 1.032 (0.916–1.163) | 0.608 | 1.030 (0.915–1.161) | 0.622 | 0.984 (0.857–1.131) | 0.823 | 0.984 (0.857–1.131) | 0.825 | 0.985 (0.856–1.133) | 0.835 |
| ECW/TBW (per 0.01 increase) | 1.052 (0.954–1.159) | 0.313 | 1.049 (0.951–1.157) | 0.340 | 1.044 (0.937–1.162) | 0.436 | 1.041 (0.935–1.160) | 0.463 | 1.042 (0.935–1.161) | 0.453 |
| VFA (per 10 cm² increase) | 1.056 (0.986–1.132) | 0.117 | 1.057 (0.987–1.132) | 0.114 | 1.064 (0.987–1.147) | 0.106 | 1.066 (0.988–1.149) | 0.099 | 1.064 (0.987–1.148) | 0.105 |
| Phase angle (per 1°) | 1.016 (0.971–1.063) | 0.492 | 1.016 (0.971–1.063) | 0.489 | 1.122 (0.943–1.335) | 0.195 | 1.123 (0.942–1.339) | 0.197 | 1.126 (0.942–1.347) | 0.192 |
| Albumin (per 1 g/L) | **0.958 (0.928–0.989)** | **0.009** | **0.956 (0.925–0.987)** | **0.006** | 0.984 (0.949–1.021) | 0.400 | 0.982 (0.946–1.018) | 0.326 | 0.994 (0.955–1.035) | 0.780 |
| NLR (per 1 unit) | 0.998 (0.984–1.013) | 0.817 | 0.998 (0.984–1.013) | 0.835 | — | — | — | — | — | — |
| log1p(CRP) | — | — | — | — | **1.292 (1.135–1.471)** | **<0.001** | **1.294 (1.136–1.474)** | **<0.001** | **1.279 (1.122–1.458)** | **<0.001** |
| Any comorbidity (HT/DM/CAD) | — | — | 1.177 (0.866–1.599) | 0.298 | — | — | 1.114 (0.793–1.565) | 0.535 | — | — |

Notes: Robustness analyses were performed by extending the main multivariable logistic regression models for nutritional risk (NRS2002 ≥3). Model A included albumin and NLR, whereas Model B included albumin and log1p-transformed CRP. “+ comorb” indicates additional adjustment for any comorbidity (HT/DM/CAD), and “+ Hb” indicates additional adjustment for hemoglobin.

**Supplementary Table S5. Standardized-effect models (per 1 SD) for nutritional risk (NRS2002 ≥3).**

| Predictor (per 1 SD increase) | Model A OR (95% CI) | P value | Model B OR (95% CI) | P value |
| --- | --- | --- | --- | --- |
| SMI | 1.052 (0.867–1.275) | 0.608 | 0.975 (0.779–1.220) | 0.823 |
| ECW/TBW | 1.095 (0.918–1.307) | 0.313 | 1.081 (0.889–1.314) | 0.436 |
| VFA | 1.199 (0.956–1.505) | 0.117 | 1.228 (0.957–1.575) | 0.106 |
| Phase angle | 1.052 (0.910–1.217) | 0.492 | 1.452 (0.826–2.555) | 0.195 |
| Albumin | **0.818 (0.704–0.950)** | **0.009** | 0.929 (0.783–1.103) | 0.400 |

Notes: ORs were estimated from multivariable logistic regression models with nutritional risk defined as NRS2002 ≥3. Continuous body composition and laboratory markers were standardized (mean=0, SD=1) and modeled per 1 SD increase. Model A included albumin and NLR; Model B included albumin and log1p-transformed CRP. All models were adjusted for age, sex, BMI, cancer group, and the same set of covariates as the corresponding main models.

**Supplementary Table S6. Interaction tests by cancer system group.**

| Interaction term | P for interaction |
| --- | --- |
| SMI × GI group | 0.576 |
| ECW/TBW × GI group | 0.181 |
| VFA × GI group | 0.115 |
| Phase angle × GI group | 0.522 |
| log1p(CRP) × GI group | 0.090 |

Notes: Interaction terms were added to multivariable logistic regression models for nutritional risk (NRS2002 ≥3) to assess effect modification by cancer system group (GI vs non-GI). P values are two-sided.


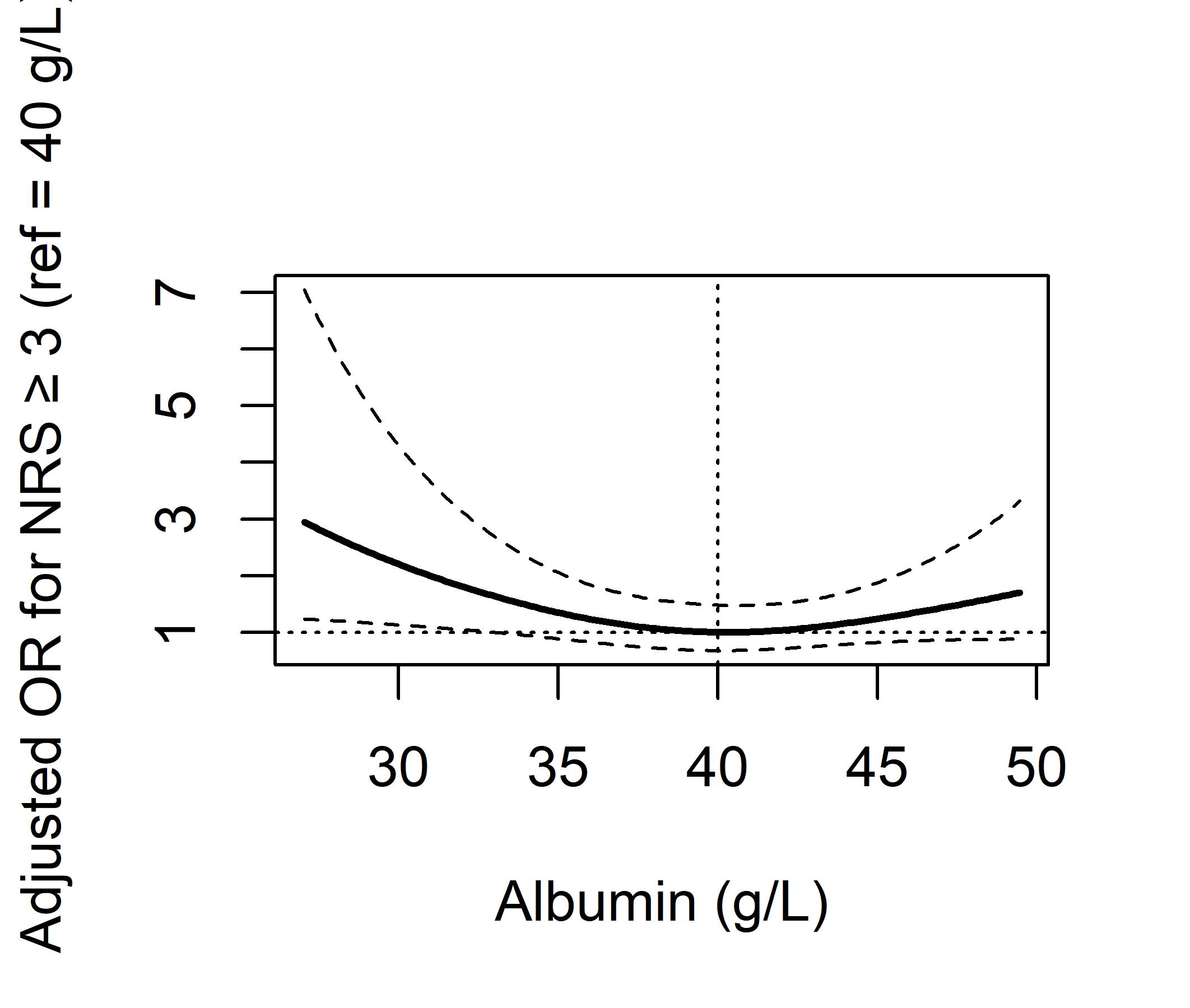


**Supplementary Figure S1. Restricted cubic spline curve for albumin and nutritional risk (NRS2002 ≥3).** Adjusted ORs and 95% CIs across albumin levels were estimated using restricted cubic splines (3 knots) from a multivariable logistic regression model. Albumin 40 g/L was used as the reference (OR=1).
